# Keeping health staff healthy: evaluation of a workplace initiative to reduce morbidity and mortality from HIV/AIDS in Malawi

**DOI:** 10.1186/1758-2652-14-1

**Published:** 2011-01-05

**Authors:** Marielle Bemelmans, Thomas van den Akker, Olesi Pasulani, Nabila Saddiq Tayub, Katharina Hermann, Beatrice Mwagomba, Winnie Jalasi, Harriet Chiomba, Nathan Ford, Mit Philips

**Affiliations:** 1 Médecins Sans Frontières - Belgium, Thyolo, Malawi; 2 Institute of Tropical Medicine, Antwerp, Belgium; 3 Thyolo District Health Office, Ministry of Health and Population, Thyolo, Malawi; 4 National Organisation of Nurses and Midwives, Malawi; 5 Médecins Sans Frontières, Cape Town, South Africa; 6 Centre for Infectious Disease Epidemiology and Research, University of Cape Town, South Africa; 7 Médecins Sans Frontières Belgium, Analysis and Advocacy Unit, Brussels, Belgium

## Abstract

**Background:**

In Malawi, the dramatic shortage of human resources for health is negatively impacted by HIV-related morbidity and mortality among health workers and their relatives. Many staff find it difficult to access HIV care through regular channels due to fear of stigma and discrimination. In 2006, two workplace initiatives were implemented in Thyolo District: a clinic at the district hospital dedicated to all district health staff and their first-degree relatives, providing medical services, including HIV care; and a support group for HIV-positive staff.

**Methods:**

Using routine programme data, we evaluated the following outcomes up to the end of 2009: uptake and outcome of HIV testing and counselling among health staff and their dependents; uptake and outcomes of antiretroviral therapy (ART) among health staff; and membership and activities of the support group. In addition, we included information from staff interviews and a job satisfaction survey to describe health workers' opinions of the initiatives.

**Results:**

Almost two-thirds (91 of 144, 63%) of health workers and their dependents undergoing HIV testing and counselling at the staff clinic tested HIV positive. Sixty-four health workers had accessed ART through the staff clinic, approximately the number of health workers estimated to be in need of ART. Of these, 60 had joined the support group. Cumulative ART outcomes were satisfactory, with more than 90% alive on treatment as of June 2009 (the end of the study observation period). The availability, confidentiality and quality of care in the staff clinic were considered adequate by beneficiaries.

**Conclusions:**

Staff clinic and support group services successfully provided care and support to HIV-positive health workers. Similar initiatives should be considered in other settings with a high HIV prevalence.

## Background

Malawi's severe health worker shortage is attributable to both an inadequate supply of trained health workers and poor retention of staff within the health system due to low remuneration, high workload, poor working conditions, illness and death [1]. Shortages of physicians and nurses are particularly acute, with only two medical doctors and 36.8 nurses per 100,000 population [2]. These levels are far below the 250 health workers per 100,000 population recommended by the World Health Organization (WHO) [3]. Numbers of other staff, including non-physician clinicians (clinical officers and medical assistants), are also insufficient [4].

In high-HIV prevalence countries such as Malawi [5] HIV/AIDS can have a negative impact on the availability of human resources in two important ways. First, HIV is a leading cause of death among health workers: one in 10 health workers in Malawi were estimated to have died of AIDS since the start of the epidemic till 1997 [6], and a study done in 1999 found an annual death rate of 2% among nursing and clinical cadres, identifying AIDS and TB as the most common causes [7]. Second, HIV leads to health workers' absence from duty by causing illness among staff themselves or among their relatives. Additional absenteeism results from health workers having to attend funerals of relatives and colleagues [8,9].

Uptake of HIV testing and counselling (HTC) and antiretroviral therapy (ART) among health workers in Malawi is low, and remained so even when these services became available in the public health system, due to the particular stigma that can be associated with being an HIV-positive health worker [10]. Studies from other high-HIV prevalence countries have highlighted the need to organize special services where staff can access a professional provider in a confidential manner [11].

In 2006, a national survey in Malawi calculated the human resource allocation providing ART in public health facilities, and concluded that the extended life years of health workers on ART exceeded health worker years needed to staff the public ART programme [12]. Other studies have reinforced this finding by proposing that the establishment of separate HIV services specifically dedicated to health workers could increase their access to essential HIV care, including ART, and in this way, would benefit the health system by reducing attrition among the health work force [13].

In Thyolo District, Malawi, the Thyolo District Health Office and Médecins Sans Frontières (MSF) established a clinic dedicated to health staff providing general medical services, including essential HIV care and a health worker support group for HIV-positive health workers. In this paper, we evaluate the essential features and outcomes of these staff health initiatives.

## Methods

### Setting

Thyolo, a rural district in the south of Malawi, has a population of approximately 600,000; the adult HIV prevalence in 2004 was 21% [14,15]. Healthcare is delivered via one large district hospital, one mission hospital, and 28 primary care facilities. Health staff ratios are lower than the national average, with only 1.3 doctors and 28 nurses per 100,000 population. In 2009, the district health office recorded 83% vacancies for clinical officers, 60% for medical assistants and 75% for nurse-midwife technicians, the most common nursing cadre [16]. As of August 2009, there were 962 health workers in the district (Table 1).

**Table 1:** Staff in Thyolo District, HMIS (August 2009)

Cadre	MoH/CHAM/tea estate clinics	MSF	VSO	Total
Medical doctor	5	1	1	**7**

Clinical officers	19	6	0	**25**

Medical assistants	36	1	0	**37**

Registered nurses	8	0	0	**8**

Nursing technicians	170	32	0	**200**

Auxiliary nurses	12	0	0	**12**

Community nurses	10	11	0	**22**

Pharmacy technicians	2	2	0	**4**

Lab technicians	4	2	0	**6**

Radiographer	2	0	0	**2**

Dental	2	0	0	**2**

Environmental officers	12	0	0	**12**

HSAs	533	0	0	**533**

Hospital attendants	83	0	0	**83**

Patient attendants	8	0	0	**8**

Total	906	55	1	**962**

Since 1997, MSF has been providing support to the district health office in the delivery of HIV/AIDS services. ART has been scaled up to district-wide access, and by the end of 2009 more than three-quarters (78%) of all patients initiated since the start of the ART programme in 2003 were alive on treatment [17].

### Programme approach

In order to overcome the barriers for health worker access to HIV services, a staff clinic was opened in July 2006 at Thyolo District Hospital. The clinic services are available for all health workers in the district and their close relatives (spouses and children). HIV services include HTC, treatment of opportunistic infections, cotrimoxazole prophylaxis, antiretroviral therapy and laboratory monitoring. The staff clinic was promoted through staff meetings, posters in the hospital and referral by the hospital support group.

In addition, the clinic provides general primary care to HIV-positive and HIV-negative health workers. This comprehensive approach aims to minimize the stigma that may arise from attending an "HIV-only" clinic [11]. General services provided include treatment of malaria, musculoskeletal problems, hypertension, diabetes, asthma and other respiratory illnesses, gastrointestinal conditions, skin diseases, and sexually transmitted infections.

Consultations are performed by a senior clinical officer accompanied by an experienced counsellor in a dedicated room within the hospital. The clinic is open every weekday from 8 am to 12 pm. All services are provided free of charge. In order to support confidentiality, staff accessing HIV services can use their own names or provide different names (their childhood names). Job titles are not recorded as this was stated in key informant interviews to be a concern.

In addition, a group of HIV-positive staff established a support group at the district hospital to provide a support network for HIV-positive health workers. The group consists of both MSF and Ministry of Health staff who organize meetings every two weeks to discuss physical, psychological and social needs. Support group members include nurses, counsellors and ward attendants.

These district initiatives - a dedicated staff clinic and a health worker support group - were the first of their kind in Malawi. Since their inception, some other districts have implemented dedicated staff health services, the majority providing HIV care alone.

### Data collection and analysis

We used a mixed-methods approach to evaluate the following outcomes: uptake and outcome of HTC among health workers and their dependents; uptake of ART among health workers only (data could not be separately extracted for dependants) and outcomes while on treatment; membership and activities of the support group; and opinions among staff about the implemented initiatives.

Patient characteristics (age, sex and CD4 count at baseline) and ART outcomes (time on ART and outcome at study end) were collected using FUCHIA software (Epicentre, Paris, France) and Microsoft Excel databases maintained by MSF for routine programme monitoring. HTC data were extracted from clinic registers from July 2006 (programme inception) to June 2009. The cumulative probability of progression to death is described using Kaplan-Meier estimates.

To evaluate health workers' opinions, we included results from a job satisfaction survey that was performed as a routine management activity in June 2009. In addition, semi-structured interviews were conducted with three key informants representing the various stakeholders pertinent to this evaluation: a clinician at the staff clinic; the chairperson of the staff support group; and the coordinator of the "Caring for Caregivers project" of the National Organisation of Nurses and Midwives in order to get the national perspective and compare with other initiatives. Qualitative data analysis of these interviews took place through extracting notes from the interviews and taking out relevant parts for this evaluation.

All data were analyzed using SPSS version 17 (New Jersey, USA).

Data were collected as part of routine programme monitoring and evaluation, and anonymized prior to being made available for analysis. The secondary analysis of routinely collected data is exempt from ethics review by both the Malawi National Health Sciences Research Committee and the MSF Independent Ethics Review Board.

## Results

Between July 2006 and June 2009, 144 clients (health workers, spouses and children) presented for HTC at the staff clinic, and of these, 91 (63%) tested HIV positive. By June 2009, 96 health workers (including 36 men) had been initiated on ART. Two-thirds (62) of them had started ART in the clinic; the rest had initiated treatment elsewhere and then self-transferred to the clinic for follow up. Of those on ART, seven staff were employed in health centres and five were from outside Thyolo District and had initiated ART in the staff clinic before returning to their respective districts for follow-up care.

The median CD4 count of staff who initiated ART in the clinic was 133 cells/mm^3^, indicating that staff presented later than the general hospital population over the same period (median CD4 count of 145 cells/mm^3^). Eight staff out of the 62 (13%) presented in an advanced stage of immune suppression with CD4 counts below 50 cells/mm^3 ^(Table 2).

**Table 2:** Baseline characteristics of the staff clinic and general ART clinic (patients who started ART)

	Staff clinic (n = 62)	General ART clinic - adults' hospital (n = 5906)
Total patients	62	5906

CD4 at initiation, median (IQR)	133 (98-222)	145 (69-234)

< 50 cells/mm^3^	(12.9%)	909 (15.4%)

< 250 cells/mm^3^	(54.8%)	3003 (50.8%)

≥250 cells/mm^3^	(17.7%)	1037 (17.6%)

Unknown	(14.5%)	957 (16.2%)

		

Female sex	35 (56.5%)	3540 (59.9%)

Age, median (IQR)	36.5 (32-41)	Specific age groups not indicated
Age groups	24-29 yrs: 11 (17.7%)	
	30-39 yrs: 29 (46.8%)	
	40-49 yrs: 17 (27.4%)	
	50-55 yrs: 5 (8.1%)	

**Figure 1:**
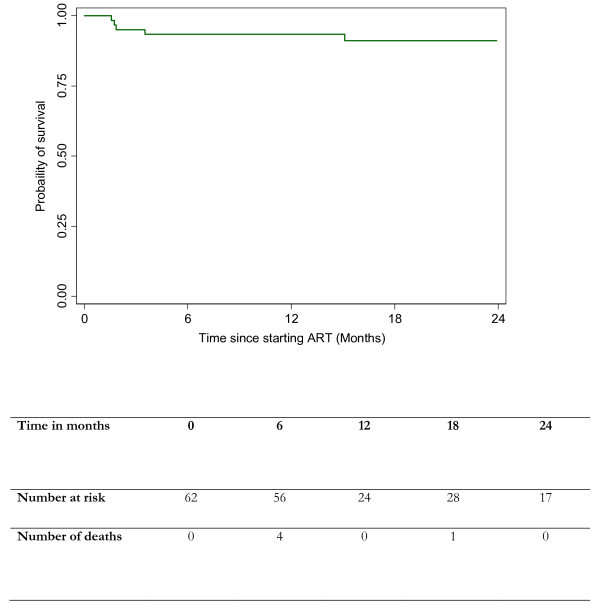
**Kaplan Meier Survival graph**.

In the outcome analysis (n = 57), we excluded the five staff who were initiated in Thyolo staff clinic but came from outside of the district. Cumulative three-year outcomes among Thyolo staff who initiated ART at the staff clinic were as follows: 91% (52/57) of Thyolo health workers who had initiated ART at the staff clinic were alive and on ART; 4% (two) had died; 5% (three) had transferred to other services in the district; and none had defaulted or stopped treatment. These outcomes are in line with the national ART programme outcomes [18]. The cumulative probability of death is described in Figure 1.

In the three years since inception, membership of the health workers' support group increased from 10 to 61, all but one of whom were still on ART at the end of the observation period (the remaining health worker is not yet eligible for ART according to clinical criteria). The majority of support group members were staff working in the district hospital with only three members from nearby health centres. According to key informant interviews with the chairperson of the support group and the clinician of the staff clinic, access from other facilities is difficult due to long travelling times.

Staff perceptions of the clinic were assessed in a job satisfaction survey conducted among 700 out of 962 health workers in Thyolo District in June 2009 as part of a routine management activity. The survey included a representative sample of all higher health workers (lower level cadres, such as ward attendants, were not included). Among all health workers, 343 responses were obtained, giving a response rate of 49%. Respondents by cadre were reflective of service distribution, apart from the laboratory and pharmacy technicians who were slightly under-represented (4.6% of staff vs. 2% of respondents).

Even though awareness of the staff clinic was high (85% at hospital level and between 73% and 79% in the peripheral centres), only between 39% (hospital staff) and 29% (health posts staff) stated they had ever made use of it. Commonly cited reasons for attending the clinic were: high quality of care, easy access and confidentiality. The most commonly reported reason for the hospital staff was ease of access; for the health centre staff the quality of care offered in the staff clinic was said to be the most important factor. The most important reasons for not attending were distance between the workplace and the clinic (especially for those working in the periphery) and inconvenient opening hours (i.e., during working time). These reasons were most frequently mentioned by staff from the peripheral sites (Table 3).

**Table 3:** Outcomes of the staff clinic related questions of the staff survey

Awareness of staff clinic	Hospital staff N = 60	Health centre staff N = 217	Health post staff N = 70	Total N = 347
Aware	52 (85%)	171 (79%)	51 (73%)	274 (79%)

Unaware	7 (11%)	39 (18%)	17 (24%)	63 (18%)

No answer	1 (2%)	7 (3%)	2 (3%)	10 (3%)

				

**Utilization of services**				

Used staff clinic at least once	24 (39%)	76 (35%)	20 (29%)	120 (35%)

Never used	33 (54%)	133 (61%)	47 (67%)	213 (61%)

No answer	3 (5%)	8 (4%)	3 (4%)	14 (4%)

				

**Reason for using staff clinic ***(surveyors could tick multiple answers)*	**Hospital** **N = 24**	**Health centre** **N = 84**	**Health post** **N = 23**	**Total** **N = 131**

Easy to access	18	38	11	67

High quality of care	2	59	12	73

Confidentiality	7	29	9	45

Friendly services	9	16	8	33

				

**Reason for NOT using staff clinic ***(surveyors could tick multiple answers)*	**Hospital** **N = 32**	**Health centre** **N = 131**	**Health post** **N = 48**	**Total** **N = 211**

Do not need services offered	7	7	1	15

Too far to access	9	88	33	130

Don't want people to know I visit staff clinic	1	3	2	6

Bad quality services	10	15	5	30

Opening hours not regular	18	52	24	94

It is an unfriendly place	5	23	5	33

According to the clinician at the staff clinic, the main perceived benefit of the staff clinic was the possibility to receive convenient ('one-stop') services in a separate room in the hospital where confidentiality is ensured by the provision of general care (and not exclusively HIV/AIDS care).

## Discussion

For high-HIV prevalence countries with human resource shortages, reducing mortality and morbidity among health workers is a critical priority. Our results show that a dedicated general staff clinic combined with an HIV support group can successfully enhance uptake of essential HIV services among health staff.

The exceptionally high HIV prevalence of 63% among those coming for HTC at the staff clinic reflects a positive self-selection bias towards those with symptoms or who suspect themselves to be positive, and reflects the acceptability of the service as a "safe place" to go for a first HIV test.

Assuming that the actual HIV prevalence among Thyolo health workers was similar to the general adult HIV prevalence in the district (21%) and that 30% of HIV-positive adults were in need of ART (based on national approximates) [5], we estimate that the expected number of staff requiring ART by mid-2009 was around 60. This suggests that most health workers who needed ART in the district had accessed treatment.

Results from the job satisfaction survey illustrate that there is high awareness of the staff clinic in Thyolo. Opening hours during working time are cited as a main reason for not making use of its services. Offering weekend opening hours could improve uptake. It is encouraging that concerns about anonymity was not seen to be an important barrier to access, suggesting that the confidentiality measures offered by the clinic, together with the fact that HIV services are provided as part of general services, are adequate. In response to the fact that distance was cited as a problem for staff working in more remote areas a smaller staff clinic is planned to open in a peripheral site.

The timely provision of ART to people in need supports their ability to work [19], and several studies have shown that providing ART to health workers is a particularly wise investment as it greatly contributes to reduced attrition [9,20]. Based on our experience and some of the problems raised during this evaluation, a number of features for a successful staff clinic can be proposed. These include: the provision of confidential but accessible rooms in close proximity to the workplace; awareness raising among health staff through staff meetings, pamphlets and posters; the allocation of dedicated staff, including a respected clinician accompanied by an experienced counsellor; the creation of an integrated clinic that provides HIV care as a part of a comprehensive package of general healthcare such that the clinic is not perceived as an HIV clinic; the establishment of satellite/mobile clinics in hard-to-reach peripheral areas; and flexible opening hours, including nights and weekends.

The allocation of staff to support a dedicated clinic for health staff in the context of human resource shortages may be considered as an additional burden to an already overstretched service. However, we believe that the relatively low resource requirements, both human (a half-time clinical officer and a counsellor for approximately one hour per week) and material (one room at the hospital) are more than adequately compensated for by the reduced waiting times, illness and mortality of health staff benefitting from the services. The majority (85%) of staff on ART have joined the support group, indicating a high acceptance and appreciation of this type of support. However, the fact that nurses are the highest qualified cadre registered at the staff clinic indicates that senior staff members face additional challenges to seeking care.

The provision of dedicated services for health staff remains limited in Malawi: only eight of Malawi's 28 districts provide staff clinic services [21,22]. Several groups have highlighted the need to boost access to HIV services for health workers [23,24]. The Caring for Caregivers programme, a five-year project run by the National Organisation of Nurses and Midwives, was established in 2006 in order to promote treatment and additional support for HIV-infected health workers [21]. Health workers who want to attend healthcare anonymously are linked to a support network that refers them to appropriate services outside of their own workplace. The Thyolo support group is linked with the national Caring for Caregivers programme of the NONM, which coordinates exchange visits with other districts in order to promote the support group concept and share lessons learnt.

Our study is subject to a number of limitations. Our analysis is based on secondary data collected for routine clinical care and, as such, we are only able to report on a limited number of variables. We chose this operational research approach in order to minimize the burden of data collection to routine services. Due to confidentiality issues, we are not able to report outcomes disaggregated by cadre. We did not undertake any formal sampling procedure for the qualitative survey so the reports will be compromised in validity. Health surveillance assistants formed 70% of the health staff included in this survey and represented 60% of the staff survey respondents; thus the overall findings of the questions of the survey may be biased towards this cadre, although this proportion is reflective of actual staffing ratios. Workers in remote locations were adequately represented (30% of survey respondents compared with 36% as actual staffing levels). However, there was a high rate of non-responses (51%) so survey results can be taken as only indicative rather than representative.

## Conclusions

A dedicated staff clinic and a health worker support group at the workplace in Thyolo District, Malawi, successfully increased the uptake of HTC and ART among health workers in the district and these initiatives were well received by clients. The investment made to staff the clinic is, we believe, more than adequately compensated for by the increase in working hours resulting from a reduction in illness and death among health staff. In this way, dedicated HIV services for health staff is an important approach to minimizing the human resource crisis in high-HIV burden settings like Malawi.

## Competing interests

The authors declare that they have no competing interests.

## Authors' contributions

MB and TvdA conceptualized the study and wrote a first draft, which was edited by all other authors. OP, WJ, NST and TvdA assisted with data collection. MB, BM and HC authorized the interventions. KH and NF checked scientific soundness and reviewed the manuscript several times. MB, TvdA, NF, MP and KH finalized the manuscript. All authors contributed considerably to the intellectual content of this article. All authors read and approved the final version prior to publication.
